# Graded Incorporation of Defatted Yellow Mealworm (*Tenebrio molitor*) in Rainbow Trout (*Oncorhynchus mykiss*) Diet Improves Growth Performance and Nutrient Retention

**DOI:** 10.3390/ani9040187

**Published:** 2019-04-23

**Authors:** Paulo Rema, Subramanian Saravanan, Benjamin Armenjon, Constant Motte, Jorge Dias

**Affiliations:** 1 CECAV (Animal and Veterinary Research Center), Universidade de Trás-os-Montes e Alto Douro, Quinta de Prados, 5001-801 Vila Real, Portugal; prema@utad.pt; 2 ŸNSECT, Genopole-Campus 3/Batiment 2–1, Rue Pierre Fontaine, 91058 Evry, France; saravanan.subramanian@skretting.com (S.S.); bar@ynsect.com (B.A.); constant.motte@ynsect.com (C.M.); 3 SPAROS LDA, Área Empresarial de Marim, 8700-221 Olhão, Portugal

**Keywords:** fishmeal, insect meal, mealworm, rainbow trout, growth performance, digestibility

## Abstract

**Simple Summary:**

Efforts to find sustainable ingredients for aquaculture feeds have been increasing. Insect meal is a promising and emerging ingredient because insects are part of the natural diet of fish and have a low ecological footprint. Here, we studied the effect of a gradual replacement of fishmeal with insect meal from yellow mealworm on juvenile rainbow trout performance. Overall, fish grew faster with the incorporation of the insect meal in the feed, and their capacity to convert feed into fish biomass also increased. The retention of ingested key nutrients also increased with the incorporation of insect meal in the feed. In summary, juvenile trout fed an insect-based diet grew faster and required lower feed intake to grow than juvenile trout fed on a common diet with standard levels of fishmeal. These results support the transition of fishmeal to insect meal in aquafeeds, which will improve the sustainability of the aquaculture industry.

**Abstract:**

Insects are emerging as a sustainable alternative to fishmeal and fish oil in aquafeeds. This study assessed the effect of graded incorporation levels of defatted yellow mealworm (*Tenebrio molitor*) protein meal on juvenile rainbow trout (*Oncorhynchus mykiss*) growth performance, body composition, and apparent nutrient digestibility. The trial comprised five dietary treatments: control diet with 25% fishmeal, and four experimental diets with yellow mealworm protein meal at 5%, 7.5%, 15%, or 25%, which corresponded to a fishmeal replacement of 20%, 30%, 60%, or 100%, respectively. After 90 days, the graded incorporation of insect protein meal led to a significant stepwise increase in final body weight, and a significant improvement of specific growth rate, feed conversion ratio, and protein efficiency ratio compared to the control treatment. Regardless of the incorporation level, the insect protein meal had no effects on fish whole-body composition and apparent digestibility coefficients of dry matter, protein, fat, phosphorus, and energy. Protein, phosphorus, and energy retention significantly increased in fish fed the diets with an insect protein meal. In conclusion, the yellow mealworm protein meal could effectively replace 100% of fishmeal in the diet of juvenile rainbow trout with positive effects on its overall zootechnical performance.

## 1. Introduction

Fishmeal (FM) has been the primary source of protein in aquafeeds due to its high protein content, balanced composition of amino acids, and high palatability. However, the increasing demand for FM and fish oil (FO) by the aquaculture industry, has led to a notable price increase of these two commodities [1]. Thus, great efforts are currently being made by the aquafeed industry to find more profitable and sustainable protein-rich ingredients [2,3]. For instance, plant-based products made of soy, corn, wheat, have been suggested as possible alternative ingredients with high protein content [4,5]. However, feed ingredients of vegetable origin have disadvantages, such as the presence of anti-nutritional factors, low content of particular essential amino acids, and low palatability [6]. More alternative ingredients are therefore needed to sustainably promote aquaculture growth while improving fish growth and health performances.

Insect meal (IM) is rich in amino acids, lipids, vitamins, and minerals, and has been suggested as a promising and natural alternative to FM and FO [2,7,8]. It is extremely rich in crude protein, which accounts for 50% to 82% of its dry mass depending on the insect species and processing method [9]. Such protein content is within the range commonly observed for FM (73% protein content), but it is superior to that of vegetable sources such as soymeal that contains only up to 50% of protein [7]. Moreover, the ecological footprint of IM production is relatively small, as there is no need for arable land, and energy and water requirements are considerably lower than those needed for other crops, roughages, and crop by-products [10]. A life cycle assessment study indicated that insect proteins present lower environmental impacts than fishmeal over most of the impact criteria [11]. Although, such beneficial features are strongly conditioned by the type of agricultural co-products and waste-streams used as nutritional substrates to grow the insects [12,13]. Given these promising features of IM, several types of insects, such as locusts, grasshoppers, termites, yellow mealworms, Asiatic rhinoceros beetles, superworms, domesticated silkworms, common houseflies, common mosquitoes, and black soldier flies have been evaluated for inclusion in aquafeeds [2].

Yellow mealworm (*Tenebrio molitor*), a member of the family Tenebrionidae, is one of the most promising insect species suitable for mass production because it is easy to breed and feed [7,14]. Mealworm larvae fed on plant by-product diets grow well [15] and have a short life cycle: the egg stage lasts 3 to 9 days, the larval stage lasts 26 to 76 days, and the pupal stage lasts 5 to 17 days [16]. Larvae of *T. molitor* are rich in protein and unsaturated fat, and processed *T. molitor* larvae meal has been tested as a replacement of FM in the diet of several aquatic species. For instance, the inclusion of *T. molitor* larvae meal in the diet of gilthead seabream (*Sparus aurata*) at 25% and 50%, which represented a 33% and 74% replacement of FM, showed no negative effects on fish growth performance [17]. Moreover, the 33% FM substitution resulted in optimal weight gain, feed conversion ratio (FCR), and protein efficiency ratio (PER), while the coefficients of apparent digestibility decreased as IM inclusion level increased [17]. Another study showed that up to 40% of FM could be replaced by IM from yellow mealworm larvae (17% incorporation level) in diets of African catfish (*Clarias gariepinus*) without affecting growth performance or FCR [18]. Juvenile rockfish (*Sebastes schlegeli*) showed no adverse health and growth performances when up to 38% of the FM in its diet was replaced by IM from mealworm larvae (32% incorporation level) [19]. Belforti et al. revealed that rainbow trout (*Oncorhynchus mykiss*) fed diets in which 33% or 66% of the FM was replaced with mealworm IM (25% and 50% incorporation level, respectively) showed no differences on fish growth, but the replacement significantly improved FCR and fish survival [14]. It is, however, important to note that most studies addressing FM replacement with IM use whole dried insect larvae, which contain a relatively high-fat level [20]. IM usually shows an excellent composition of essential amino acids, but a low-quality fatty acid profile [2]. Defattening IM is a method that can improve its palatability and nutrient digestibility, thereby making it a more suitable and protein-rich ingredient for fish diets [2]. Nonetheless, the effect of replacing FM with insect protein meal (IPM) from defatted mealworm on fish performance is still poorly investigated [2].

The present study assessed the effect of dietary inclusion of defatted yellow mealworm protein meal on juvenile rainbow trout (*O. mykiss*) zootechnical performance, whole-body composition, and nutrient digestibility. Increasing replacement levels of dietary FM (from 20% to 100%) with IPM were tested to assess if this insect-based protein source has the potential for full replacement of FM in juvenile rainbow trout diet.

## 2. Materials and Methods

The trial was conducted at the experimental research station of the University of Trás-os-Montes e Alto Douro (UTAD, Portugal). Experiments were directed by trained scientists (following category C FELASA recommendations) and in compliance with the European (Directive 2010/63/EU) and Portuguese (Decreto-Lei n 113/2013, de 7 de Agosto) legislation on the protection of animals for scientific purposes. UTAD facilities and their staff are certified to house and conduct experiments with live animals (‘group-1’ license by the ‘Direção Geral de Veterinária’, Ministry of Agriculture, Rural Development and Fisheries of Portugal).

### 2.1. Test Ingredient and Experimental Diets

A fishmeal-based control diet (CTRL) comprising 25% FM, 8% of squid meal and krill meal, soy protein concentrate, wheat gluten, and corn gluten was formulated with practical ingredients to fulfill the known nutritional requirements of rainbow trout (*O. mykiss*) juveniles. Based on this formulation, four test diets (IPM5, IPM7.5, IPM15, and IPM25) were formulated (Table 1), in which FM was replaced at 20%, 30%, 60%, and 100%, respectively, by the yellow mealworm protein meal (the IPM tested here) obtained by processing the larvae of *T. molitor* reared on complete plant by-products at Ynsect (Dole, France). Proximate composition, and the amino acid and fatty acid profiles of the yellow mealworm protein meal are shown in Table S1. Squid and krill meal levels were maintained constant in all diets to guarantee high palatability. Test diets required minor adjustments on their formulations to maintain isonitrogenous [crude protein, 48.5% dry matter (DM)], isolipidic (22.7% DM), and isoenergetic (gross energy, 23.2 MJ/kg DM) conditions. Methionine and monocalcium phosphate supplementation were adjusted in test diets to match that of the CTRL diet.

All powder ingredients were mixed at SPAROS (Olhão, Portugal) according to the target formulation in a 500-L double-helix mixer (TGC Extrusion, Roullet-Saint-Estèphe, France) and ground (<400 µm) in a SH1 micropulverizer (Hosokawa-Alpine, Augsburg, Germany). Diet pellets (1.2 and 2.0 mm) were then manufactured by extrusion and dried in a DR100 vibrating fluid bed dryer (TGC Extrusion). After cooling, oils were added using the PG-10VCLAB vacuum coating device (Dinnissen, Sevenum, The Netherlands). Throughout the trial, experimental feeds were stored in a cool and aerated chamber at room temperature. Samples of each diet were taken for detailed analyses of amino acid and fatty acid profiles (Table S2).

### 2.2. Growth Performance Trial

Triplicate groups of 35 juvenile rainbow trout with a mean initial body weight (IBW) of 5.01 ± 0.1 g were fed one of the five experimental diets during 90 days. Fish were grown in 250-L fiberglass circular tanks (initial stocking density of 0.7 kg/m^3^) supplied with flow-through freshwater at 14.1 ± 0.3 °C and dissolved oxygen levels above 7.4 mg/L, under natural photoperiod changes from May to July. Fish were hand-fed to apparent satiety two or three times per day (at 9.00, 14.00, and 18.00 on weekdays; at 10.00 and 16.00 on weekends). Distributed feed was quantified throughout the trial. Anesthetized fish were individually weighed at the start and at the end of the trial. At the beginning of the trial, 15 fish from the same initial stock were sampled and stored at −20 °C for subsequent whole-body composition analysis. At the end of the trial, six fish from each tank were sampled for the same purpose.

### 2.3. Apparent Digestibility

At the end of the growth performance trial, 12 fish (body weight: ca. 45 g) from each tank were used to determine the apparent digestibility of dry matter, protein, fat, energy, and phosphorus, following the indirect method with identical diets containing yttrium oxide (Y_2_O_3_, 200 mg/kg) as the inert tracer. Fish were stocked in 60-L cylindro-conical tanks with flow-through (3.7 L/min) freshwater at 14 °C and dissolved oxygen levels above 6.4 mg/L, and acclimated for 10 days to rearing conditions and experimental diets. After this period, fish were hand-fed in slight excess daily (at 10.00). Upon a thorough cleaning of the rearing tanks to remove any feed residues, feces were collected daily for the following eight days, using the continuous outlet water filtration system (Choubert-INRA system), and kept at −20 °C. Pooled feces from the fish within each tank were freeze-dried before analysis. Each dietary treatment was tested in triplicate.

Apparent digestibility coefficients (ADC) of dietary nutrients and energy were calculated for each experimental diet as:

(1) $$\mathrm{ADC}\;\left(\%\right)=100-\;\left[\frac{{\%\;\mathrm{dietary}\;Y}_{2}O_{3}\;\mathrm{level}}{{\%\;\mathrm{feacal}\;Y}_{2}O_{3}\;\mathrm{level}}\;\times \;\frac{\%\;\mathrm{faecal}\;\mathrm{nutrient}\;\mathrm{or}\;\mathrm{energy}\;\mathrm{level}}{\%\;\mathrm{dietary}\;\mathrm{nutrient}\;\mathrm{or}\;\mathrm{energy}\;\mathrm{level}}\right]$$

### 2.4. Chemical Composition Analyses

The IPM alone, five experimental diets, and freeze-dried feces were ground. Fish whole-body samples from each tank were minced, mixed, and a representative sample was freeze-dried and homogenized. These ground/homogenized samples were then analyzed according to methodologies described by AOAC [21]. The DM content was determined after drying samples at 105 °C for 24 h and ash content was determined by combustion at 550 °C for 12 h. Crude protein content (N × 6.25) was determined by the flash combustion technique followed by gas chromatographic separation and thermal conductivity detection on an FP 428 elemental analyzer (LECO Corp., Saint Joseph, MI, USA). Fat content was determined by dichloromethane extraction on a Soxhlet apparatus. Total phosphorus was measured according to the ISO 6491:1998 [22] method using the vanado-molybdate reagent. Gross energy was determined in an adiabatic bomb calorimeter (IKA-Werke GmbH & Co. KG, Staufen, Germany). Yttrium oxide in feeds and feces was determined by inductively coupled plasma atomic emission spectroscopy (ICP-AES) according to the methodology described by Reis et al. 2008 [23].

Total amino acids in the IPM alone and experimental diets were hydrolyzed (6 M HCl at 116 °C over 22 h in nitrogen-flushed glass vials) and then pre-column derivatized with the AccQ Fluor Reagent (6-aminoquinolyl-N-hydroxysuccinimidyl carbamate) using the AccQ Tag method (both Waters, Milford, MA, USA) [24]. Ultra-high-performance liquid chromatography (UPLC) was then performed in a reversed-phase amino acid analysis system (Waters) using norvaline as the internal standard. Tryptophan was not determined because it is partially destroyed by acid hydrolysis. The resultant peaks were analyzed with EMPOWER software (Waters).

For fatty acid analysis, lipids were first extracted according to the method of Folch et al. [25]. The fatty acid composition of fish fillets was determined by gas-chromatography analysis of methyl esters, according to the procedure of Lepage and Roy [26].

### 2.5. Growth and Nutrient Utilization

To evaluate fish growth and nutrient utilization, the following indices were calculated:

- Specific growth rate (SGR; %/day) = (Ln FBW − Ln IBW) × 100, where FBW and IBW are the final and initial body weight of fish (g), respectively;
- Feed conversion ratio (FCR) = crude feed intake (g)/weight gain (g);
- Feed intake (FI; %BW/day) = crude feed intake (g)/((IBW + FBW)/2/days) × 100;
- Protein efficiency ratio (PER) = wet weight gain (g)/crude protein intake (g);
- Retention (% of intake): 100 × (FBW × final carcass total nutrient content − IBW × initial total carcass nutrient content)/nutrient intake.

### 2.6. Statistical Analyses

Data are presented as means of three replicates and standard deviations. After arcsine square root transformation of the data obtained as percentages, all data were subjected to a one-way analysis of variance (ANOVA) considering *p* ≤ 0.05 as significant. All statistical tests were performed in SPSS v. 21 (IBM, Armonk, NY, USA).

## 3. Results

### 3.1. Growth Performance

At the end of the 90-days of experimental feeding, fish from the best performing treatment (IPM25) showed an 11-fold increase of their IBW (Table 2). Fish fed the insect-rich diets showed a significant increase of final body weight compared to CTRL fish (FBW, *p* < 0.05, *F* = 65.959, df = 14). This increase was dose-dependent, with a moderate increase for IPM5, intermediate increase for IPM7.5 and IPM15, and highest increase for IPM25. The SGR was lowest in fish fed the CTRL diet, while those fed diets containing IPM showed significantly higher SGR values (*p* < 0.05, *F* = 18.115, df = 14). Irrespective of the incorporation level, IPM diets led to a significant reduction of FCR (*p* < 0.05, *F* = 12.427, df = 14). In comparison to the CTRL treatment, all IPM diets led to a significant reduction of feed intake and a significant increase of PER values (*p* < 0.05, *F* = 9.212, df = 14).

### 3.2. Whole-Body Composition

Fish whole-body composition was not different across the five experimental treatments (Table 3). There were also no significant differences amongst experimental diets in terms of moisture, protein, fat, ash, and phosphorus percentages, as well as in energy levels (Table 1).

### 3.3. Nutrient Retention

Nutrient and energy retention (expressed as percentage of intake) generally increased with the incorporation of IPM in the rainbow trout diet (Table 4). In comparison to the CTRL group, all IPM-fed groups showed a significant increase in the retention of protein, phosphorus, and energy (*p* < 0.05, *F*_(protein)_ = 7.497, *F*_(phosphorus)_ = 3.929, *F*_(energy)_ = 5.530). Similarly, diets IPM7.5, IPM15, and IPM25 showed a significantly higher phosphorus retention than the CTRL diet (*p* < 0.05). The retention of fat was not affected by dietary treatments (*p* = 0.32, *F* = 1.343).

### 3.4. Apparent Digestibility

The ADC for the various nutrients and energy are presented in Table 5. The increasing incorporation levels of IPM had no significant effect (*p* > 0.05) on the apparent digestibility of DM, protein, fat, phosphorus, and energy.

## 4. Discussion

The replacement of FM with IPM has been previously assessed for rainbow trout. Particularly, Belforti et al. [14] used a commercial full-fat insect meal from *T. molitor* larvae, whereas Renna et al. [27] used a partially defatted black soldier fly (*Hermetia illucens*) larvae meal. Both studies evidenced that IPM could be used to replace FM in rainbow trout diets. Moreover, both studies identified a poor lipid profile of fish fed IPM, which could be associated with the poor fatty acid profile of the tested ingredients [2]. To our best knowledge, the present study is the first to address the use of fully defatted IPM to partially or fully replace FM in rainbow trout diet.

The present results show that replacement of FM with fully defatted IPM improves growth performance to the highest range observed for juvenile rainbow trout [27,28,29,30]. For example, SGR varied between 2.4%/day and 2.7%/day, which is almost twice the SGR observed for rainbow trout fed a fully-fat *T. molitor* insect meal, regardless of the replacement level [14], and also higher than the results observed with black soldier fly insect meal, which averaged 1.41% [27]. In the present study, FCR varied between 0.79 and 0.93 among treatments, indicating the good nutritional adequacy of the feeds and good feeding practices (Table 2). The significant stepwise increase in zootechnical parameters (Table 2), suggests the positive role of defatted IPM on the growth performance of rainbow trout even at full FM replacement. It is, however, important to note that optimal FM replacement levels often vary with fish species and the defattening level of the IPM. For instance, *S. aurata* optimal body-weight gain is achieved when FM is replaced at 25% with full-fat *T. molitor* insect meal [17]. Similar results were obtained for African catfish, where a 20% substitution with full-fat mealworm insect meal improved fish growth performance, but negative performance results were observed if replacement levels exceeded 20% [18]. The significant reduction of feed intake observed in the present study for fish fed on IPM has also been reported for rainbow trout diets with FM replaced with insect meal [14]. While the latter study attributed the reduction of voluntary feed intake to the high-fat content of the whole-dry-insect meal, this does not apply to the present study where defatted IPM was used. Although the presence of anti-nutritional factors in the IPM used in the present study were not investigated, we hypothesize that the reduction of feed intake observed is probably linked to a better nutritional and metabolic adequacy of diets rather than stress or feed palatability, as indicated by the significantly lower FCR and higher growth performance (Table 2).

In line with previous studies [19,31], there were no differences in the body composition of rainbow trout fed with the five experimental diets (Table 3). This result supports the partial or full replacement of FM with IPM without adversely affecting moisture, protein, fat, ash, and phosphorus contents of rainbow trout. This is a notable achievement for the future application of this IPM in rainbow trout aquafeeds, as previous studies have shown notable changes in fish whole-body or fillet quality with the inclusion of insect meal in fish diets. For instance, the whole-body fat composition of African catfish was notably altered when a diet with a low level of insect meal (8%) was used [18]. European sea bass showed no whole-body composition changes at 25% insect meal dietary inclusion, but at 50% inclusion level significant changes were observed in ash content. It is possible that the positive results observed for juvenile rainbow trout are associated with the use of defatted IPM and with the significant increases in the retention of protein, phosphorus, and energy in IPM diets (Table 4). The similar and relatively high ADC of the various nutrients and energy observed for rainbow trout fed IPM diets (Table 5) suggest that the IPM used in the present study is well digested by rainbow trout, which might contribute to the positive whole-body composition, nutrient retention, and growth performance results, as observed in previous studies [14,27,32]. The similar ADC results among experimental diets and the amino acid and fatty acid compositions of the IPM diets tested here also support the highly efficient metabolic utilization of the nutrients observed here.

## 5. Conclusions

In conclusion, the defatted yellow mealworm protein meal tested in the present study could effectively replace up to 100% of the FM in the diet of juvenile rainbow trout with positive zootechnical and nutrient retention consequences. It is particularly important to highlight that the best growth performance and FCR were recorded when FM was fully replaced by IPM, supporting that this IPM is a sustainable and environmentally friendly source of protein in the diet of rainbow trout. Additionally, the potential effects of IPM on fish immunity and health status are worth further research.

## Figures and Tables

**Table 1:** Formulation and proximate composition of the experimental diets (Control: CTRL, and test diets with 5%, 7.5%, 15%, or 25% insect protein meal (IPM), respectively IPM5, IPM7.5, IPM15, and IPM25).

Ingredients (%)	CTRL	IPM5	IPM7.5	IPM15	IPM25
Fishmeal LT70 ^1^	25.00	20.00	17.50	10.00	0.00
Krill meal ^2^	3.00	3.00	3.00	3.00	3.00
Squid meal ^3^	5.00	5.00	5.00	5.00	5.00
Yellow mealworm protein meal ^4^		5.00	7.50	15.00	25.00
Soy protein concentrate ^5^	14.00	14.00	14.00	14.00	14.00
Wheat gluten ^6^	9.05	9.25	9.40	9.65	10.10
Corn gluten ^7^	8.20	8.20	8.20	8.20	8.20
Soybean meal 48	7.50	7.50	7.50	7.50	7.50
Whole peas	6.15	5.75	5.40	4.75	3.70
Fish oil	11.50	11.50	11.50	11.50	11.50
Rapeseed oil	6.00	5.80	5.70	5.40	5.00
Vitamin & Mineral premix ^8^	1.50	1.50	1.50	1.50	1.50
Soy lecithin	1.00	1.00	1.00	1.00	1.00
Guar gum	0.20	0.20	0.20	0.20	0.20
Antioxidant	0.20	0.20	0.20	0.20	0.20
Sodium propionate	0.10	0.10	0.10	0.10	0.10
MCP	1.30	1.70	2.00	2.60	3.50
DL-Methionine	0.30	0.30	0.30	0.40	0.50
Yttrium oxide ^9^	0.02	0.02	0.02	0.02	0.02
Proximate composition					
Dry matter (DM, %)	93.4 ± 0.0	93.1 ± 0.0	93.2 ± 0.1	95.0 ± 0.0	93.2 ± 0.0
Crude protein (%DM)	48.5 ± 0.0	48.5 ± 0.1	48.5 ± 0.0	48.5 ± 0.0	48.5 ± 0.1
Crude fat (%DM)	22.7 ± 0.2	22.7 ± 0.1	22.6 ± 0.2	22.7 ± 0.2	22.7 ± 0.2
Ash (%DM)	9.4 ± 0.0	8.8 ± 0.0	8.7 ± 0.1	8.1 ± 0.0	7.4 ± 0.0
Total phosphorus (%DM)	1.4 ± 0.0	1.4 ± 0.0	1.4 ± 0.0	1.4 ± 0.0	1.4 ± 0.0
Gross energy (MJ/kg DM)	23.2 ± 0.2	23.2 ± 0.0	23.2 ± 0.0	23.2 ± 0.1	23.2 ± 0.1
Yttrium oxide (mg/kg DM)	219.0 ± 4.2	219.5 ± 6.4	222.0 ± 4.1	219.0 ± 2.8	221.0 ± 4.2

^1^ Peruvian fishmeal LT70: 71% crude protein (CP), 11% crude fat (CF), EXALMAR, Peru; ^2^ Krill meal: 61% CP, 19% CF, Aker BioMarine Antarctic AS, Norway; ^3^ Super Prime without guts: 82% CP, 3.5% CF, Sopropêche, France; ^4^ Yellow mealworm protein meal: 67.1% CP, 13.6, Ynsect, France. ^5^ Soycomil P: 62% CP, 0.7% CF, ADM, The Netherlands; ^6^ VITEN: 84.7% CP, 1.3% CF, ROQUETTE, France; ^7^ Corn gluten meal: 61% CP, 6% CF, COPAM, Portugal; ^8^ PREMIX Lda, Portugal. Vitamins (IU or mg/kg diet): DL-alpha tocopherol acetate, 100 mg; sodium menadione bisulphate, 25 mg; retinyl acetate, 20,000 IU; DL-cholecalciferol, 2000 IU; thiamin, 30 mg; riboflavin, 30 mg; pyridoxine, 20 mg; cyanocobalamin, 0.1 mg; nicotinic acid, 200 mg; folic acid, 15 mg; ascorbic acid, 1000 mg; inositol, 500 mg; biotin, 3 mg; calcium pantothenate, 100 mg; choline chloride, 1000 mg, betaine, 500 mg. Minerals (g or mg/kg diet): cobalt carbonate, 0.65 mg; copper sulphate, 9 mg; ferric sulphate, 6 mg; potassium iodide, 0.5 mg; manganese oxide, 9.6 mg; sodium selenite, 0.01 mg; zinc sulphate, 7.5 mg; sodium chloride, 400 mg; calcium carbonate, 1.86 g; excipient wheat middlings; ^9^ Sigma-Aldrich.

**Table 2:** Growth performance of rainbow trout fed the five experimental diets over 90 days (Control: CTRL, and experimental diets with 5%, 7.5%, 15%, or 25% insect protein meal (IPM), respectively IPM5, IPM7.5, IPM15, and IPM25). Values are means ± standard deviations (*n* = 3). Different superscript letters indicate significant differences (*p* < 0.05) between experimental treatments.

Dietary Treatments	CTRL	IPM5	IPM7.5	IPM15	IPM25
IBW ^1^ (g)	5.0 ± 0.1	4.9 ± 0.1	5.0 ± 0.1	5.1 ± 0.1	5.1 ± 0.1
FBW ^2^ (g)	42.9 ± 1.3 ^a^	45.2 ± 1.0 ^b^	49.0 ± 0.6 ^c^	51.0 ± 1.4 ^c^	55.9 ± 1.0 ^d^
SGR ^3^ (%/d)	2.39 ± 0.06 ^a^	2.47 ± 0.02 ^b^	2.54 ± 0.03 ^b^	2.56 ± 0.05 ^b^	2.67 ± 0.04 ^c^
FCR ^4^	0.93 ± 0.02 ^b^	0.83 ± 0.03 ^a^	0.80 ± 0.02 ^a^	0.79 ± 0.04 ^a^	0.79 ± 0.02 ^a^
Feed intake (%BW/d)	1.63 ± 0.04 ^b^	1.48 ± 0.05 ^a^	1.45 ± 0.04 ^a^	1.44 ± 0.07 ^a^	1.47 ± 0.05 ^a^
PER ^5^	2.38 ± 0.06 ^a^	2.68 ± 0.10 ^b^	2.76 ± 0.06 ^b^	2.80 ± 0.15 ^b^	2.74 ± 0.08 ^b^

^1^ IBW: Initial body weight; ^2^ FBW: Final body weight; ^3^ SGR: Specific growth rate; ^4^ FCR: Feed conversion ratio; ^5^ PER: Protein efficiency ratio.

**Table 3:** Whole-body composition of rainbow trout fed the five experimental diets (Control: CTRL, and experimental diets with 5%, 7.5%, 15%, or 25% insect protein meal (IPM), respectively IPM5, IPM7.5, IPM15, and IPM25). Values are means ± standard deviations (*n* = 3).

Body Composition ^1^	CTRL	IPM5	IPM7.5	IPM15	IPM25
Moisture (%)	70.1 ± 0.6	70.7 ± 0.4	71.1 ± 0.4	70.5 ± 0.5	70.7 ± 1.2
Protein (%)	14.8 ± 0.6	14.8 ± 0.3	15.0 ± 0.5	15.2 ± 0.3	15.2 ± 0.7
Fat (%)	12.2 ± 0.2	11.5 ± 0.4	11.0 ± 0.3	11.6 ± 0.1	11.8 ± 0.9
Ash (%)	1.9 ± 0.0	2.2 ± 0.2	2.1 ± 0.3	2.1 ± 0.0	2.2 ± 0.1
Phosphorus (%)	0.4 ± 0.0	0.4 ± 0.0	0.4 ± 0.0	0.4 ± 0.0	0.4 ± 0.0
Energy (kJ/g)	8.2 ± 0.1	8.0 ± 0.0	8.0 ± 0.0	8.0 ± 0.2	8.2 ± 0.4

^1^ Initial body composition: moisture 75.0%, protein 14.1%, fat 8.7%, ash 2.2%, phosphorus 0.4%, and energy 6.7 kJ/g.

**Table 4:** Nutrient and energy retention in rainbow trout fed the five experimental diets (Control: CTRL, and experimental diets with 5%, 7.5%, 15%, or 25% insect protein meal (IPM), respectively IPM5, IPM7.5, IPM15, and IPM25). Values are means ± standard deviations (*n* = 3). Different superscript letters indicate significant differences (*p* < 0.05) between experimental treatments.

Retention (% Intake)	CTRL	IPM5	IPM7.5	IPM15	IPM25
Protein	35.5 ± 2.5 ^a^	39.8 ± 0.7 ^b^	41.6 ± 0.4 ^b^	42.8 ± 2.2 ^b^	41.9 ± 2.2 ^b^
Fat	64.4 ± 2.1	68.0 ± 4.9	66.8 ± 3.3	71.5 ± 3.4	70.9 ± 6.7
Phosphorus	30.5 ± 0.7 ^a^	32.7 ± 1.8 ^ab^	34.0 ± 0.7 ^b^	33.9 ± 1.7 ^b^	33.8 ± 1.1 ^b^
Energy	42.0 ± 0.8 ^a^	45.4 ± 1.6 ^b^	47.1 ± 1.4 ^b^	47.8 ± 1.8 ^b^	48.0 ± 2.9 ^b^

**Table 5:** Apparent digestibility coefficients (ADC) of nutrients and energy in rainbow trout fed the five experimental diets (Control: CTRL, and experimental diets with 5%, 7.5%, 15%, or 25% insect protein meal (IPM), respectively IPM5, IPM7.5, IPM15, and IPM25). Values are means ± standard deviations (*n* = 3).

ADC (%)	CTRL	IPM5	IPM7.5	IPM15	IPM25
Dry matter	84.2 ± 0.4	84.2 ± 1.0	84.3 ± 0.7	84.0 ± 0.7	84.3 ± 0.5
Protein	93.6 ± 0.2	93.6 ± 0.4	93.6 ± 0.2	93.5 ± 0.4	93.8 ± 0.1
Fat	97.0 ± 0.1	97.0 ± 0.1	97.0 ± 0.2	97.0 ± 0.2	97.1 ± 0.3
Phosphorus (% intake)	69.9 ± 1.4	68.3 ± 1.5	70.5 ± 2.4	71.4 ± 2.9	70.3 ± 1.8
Energy (% intake)	84.1 ± 0.4	84.3 ± 0.8	84.1 ± 1.0	84.2 ± 0.6	84.4 ± 0.6

## Supplementary Materials

The following are available online at https://www.mdpi.com/2076-2615/9/4/187/s1, Table S1: Proximate composition and amino acid and fatty acid profiles of the yellow mealworm protein meal, Table S2: Amino acid and fatty acid profiles of each experimental diet.
