# Supplementary material for: Graded Incorporation of Defatted Yellow Mealworm (Tenebrio molitor) in Rainbow Trout (Oncorhynchus mykiss) Diet Improves Growth Performance and Nutrient Retention
Source: Animals (Basel). 2019 Apr 23;9(4):187. doi: 10.3390/ani9040187 (PMC6523711; doi:10.3390/ani9040187)
Supplement: Supplementary file 1 [file animals-09-00187-s001.pdf]

Table 1. Proximate composition and amino acid and fatty acid profiles of the yellow mealworm protein meal (IPM) and fishmeal (FM) (expressed as % or MJ/kg of fresh product). SFA (saturated fatty acids), MUFA (monounsaturated fatty acids), PUFA (polyunsaturated fatty acids).

**Proximate**	**Unit**	**IPM**	**FM**	**Fatty acids**	**Unit**	**IPM**	**FM**
Moisture	%	5.32	6.62	C12:0	%	0.06	0.00
Crude protein	%	67.09	71.44	C14:0	%	0.42	0.34
Crude fat	%	13.6	6.9	C16:0	%	2.24	1.52
Crude ash	%	3.21	12.74	C18:0	%	0.36	0.22
Total phosphorus	%	0.75	1.68	Other SFA	%	0.09	0.04
Energy	MJ/kg	23.74	19.68				
				C16:1n-7	%	0.58	0.39
**Essential amino acids**	**Unit**	**IPM**	**FM**	C18:1n-7	%	0.04	0.23
Arginine	%	2.56	4.73	C18:1n-9	%	5.26	1.12
Histidine	%	1.39	1.71	Other MUFA	%	0.07	0.06
Isoleucine	%	2.11	2.49				
Leucine	%	3.99	5.08	C18:2n-6	%	4.13	0.16
Lysine	%	3.32	6.03	C18:3n-3	%	0.17	0.73
Threonine	%	1.87	3.52	C20:5n-3	%	0.00	0.66
Valine	%	2.91	3.21	C22:6n-3	%	0.00	0.97
Methionine	%	1.43	2.58	Other PUFA	%	0.03	0.21
Cysteine	%	0.63	0.33				
Phenylalanine	%	1.98	3.37				
Tyrosine	%	2.68	2.58				
**Dispensable amino acids**	**Unit**	**IPM**	**FM**				
Aspartic acid + Asparagine	%	4.51	6.92				
Glutamic acid + Glutamine	%	6.36	8.92				
Alanine	%	3.83	4.38				
Glycine	%	2.54	5.20				
Proline	%	3.18	2.92				
Serine	%	2.94	3.15				

Table 2. Amino acid and selected fatty acid profile (expressed as % fed feed) of each experimental diet (Control: CTRL, and test diets with 5, 7.5, 15, or 25% insect protein meal (IPM), respectively IPM5, IPM7.5, IPM15, and IPM25).

**Amino acids**	**Unit**	**CTRL**	**IPM5**	**IPM7.5**	**IPM15**	**IPM25**
Arginine	%	4.62 ± 0.23	4.53 ± 0.02	4.49 ± 0.23	4.27 ± 0.09	3.89 ± 0.09
Histidine	%	1.47 ± 0.11	1.56 ± 0.02	1.54 ± 0.09	1.46 ± 0.07	1.50 ± 0.08
Isoleucine	%	2.31 ± 0.01	2.52 ± 0.01	2.53 ± 0.01	2.46 ± 0.02	2.49 ± 0.00
Leucine	%	4.51 ± 0.08	4.44 ± 0.01	4.68 ± 0.05	4.46 ± 0.02	4.56 ± 0.01
Lysine	%	3.09 ± 0.19	3.09 ± 0.01	3.02 ± 0.17	2.94 ± 0.01	2.97 ± 0.03
Threonine	%	2.32 ± 0.03	2.37 ± 0.00	2.31 ± 0.03	2.14 ± 0.05	2.15 ± 0.02
Valine	%	2.75 ± 0.00	2.87 ± 0.02	3.00 ± 0.03	3.08 ± 0.01	3.18 ± 0.01
Methionine	%	1.71 ± 0.15	1.71 ± 0.01	1.75 ± 0.06	1.74 ± 0.02	1.63 ± 0.02
Cysteine	%	0.35 ± 0.02	0.34 ± 0.00	0.31 ± 0.02	0.33 ± 0.00	0.34 ± 0.00
Phenylalanine	%	3.30 ± 0.00	3.06 ± 0.01	2.92 ± 0.15	2.85 ± 0.01	2.56 ± 0.00
Tyrosine	%	2.44 ± 0.11	2.48 ± 0.00	2.67 ± 0.14	2.92 ± 0.04	3.14 ± 0.12
**Fatty acids**	**Unit**	**CTRL**	**IPM5**	**IPM7.5**	**IPM15**	**IPM25**
C14:0	%	0.40 ± 0.00	0.40 ± 0.00	0.38 ± 0.00	0.43 ± 0.00	0.38 ± 0.00
C16:0	%	1.86 ± 0.01	1.89 ± 0.01	1.82 ± 0.02	2.11 ± 0.01	1.94 ± 0.02
C16:1n-7	%	0.48 ± 0.00	0.48 ± 0.00	0.44 ± 0.00	0.50 ± 0.00	0.42 ± 0.01
C18:0	%	0.49 ± 0.00	0.50 ± 0.01	0.47 ± 0.01	0.54 ± 0.00	0.50 ± 0.01
C18:1n-9	%	1.62 ± 0.01	1.74 ± 0.01	1.69 ± 0.01	2.08 ± 0.01	2.06 ± 0.02
C18:1n-7	%	0.26 ± 0.00	0.25 ± 0.00	0.23 ± 0.00	0.25 ± 0.00	0.21 ± 0.00
C18:2n-6	%	0.79 ± 0.00	0.94 ± 0.01	1.05 ± 0.01	1.36 ± 0.01	1.53 ± 0.02
C18:3n-3	%	0.13 ± 0.00	0.13 ± 0.00	0.13 ± 0.00	0.14 ± 0.00	0.12 ± 0.00
C18:4n-3	%	0.10 ± 0.00	0.10 ± 0.00	0.09 ± 0.00	0.10 ± 0.00	0.08 ± 0.00
C20:1n-9	%	0.20 ± 0.00	0.19 ± 0.00	0.17 ± 0.00	0.18 ± 0.00	0.14 ± 0.00
C20:4n-6	%	0.14 ± 0.00	0.13 ± 0.00	0.12 ± 0.00	0.14 ± 0.00	0.12 ± 0.00
C20:5n-3	%	0.72 ± 0.00	0.71 ± 0.01	0.65 ± 0.00	0.70 ± 0.00	0.57 ± 0.01
C22:1n-11	%	0.14 ± 0.00	0.13 ± 0.00	0.11 ± 0.00	0.12 ± 0.00	0.08 ± 0.00
C22:5n-3	%	0.14 ± 0.00	0.13 ± 0.00	0.12 ± 0.00	0.13 ± 0.00	0.10 ± 0.00
C22:6n-3	%	1.45 ± 0.01	1.44 ± 0.01	1.33 ± 0.01	1.46 ± 0.01	1.21 ± 0.02
